# Labor Induction with Intravaginal Misoprostol versus Spontaneous Labor: Maternal and Neonatal Outcomes

**DOI:** 10.1155/2022/2826927

**Published:** 2022-12-09

**Authors:** Esra Ozbasli, Melis Canturk, Elif Ganime Aygun, Selin Ozaltin, Mete Gungor

**Affiliations:** ^1^Acibadem Mehmet Ali Aydinlar University School of Medicine, Acibadem Maslak University Hospital, Department of Obstetrics and Gynecology, Darüşşafaka, Büyükdere Cad. No: 40, 34457 Sarıyer-Istanbul, Turkey; ^2^Unye State Hospital, Department of Obstetrics and Gynecology, Kaladere, Yuceler, Kayalar Sk.No:3, 52300 Unye-Ordu, Turkey; ^3^Acibadem Atakent University Hospital, Department of Obstetrics and Gynecology, Halkali Merkez, Turgut Ozal Blv. No: 16, Kucukcekmece- Istanbul, Turkey

## Abstract

**Purpose:**

To compare the maternal and neonatal outcomes of pregnant women who had labor induction with intravaginal misoprostol or had spontaneous labor in our clinic. *Material-Method*. The records of 213 pregnant women, who were followed up in Acibadem Maslak University Hospital for vaginal delivery between June 2021 and December 2021, were retrospectively evaluated. The pregnant women, who gave birth, were divided into 3 groups as follows: spontaneous labor (SL), those induced by a single dose of misoprostol (SDM), and those induced by multiple doses of misoprostol (MDM). The groups were compared in terms of delivery type, the vaginal birth rate within 12 hours, need for intervention, duration of the second stage of labor, cesarean section ratio due to fetal distress, time from the last dose to delivery, and 1st and 5th minute APGAR scores.

**Results:**

Among the primiparous pregnant women, 84.7% of SL group, 65.2% of SDM group, and 37% MDM group delivered vaginally within 12 hours (*p* < 0.05). The time from the last misoprostol dose to delivery was also statistically significantly shorter in pregnant women, who received a single dose of misoprostol (483 vs. 720 min, respectively). When the hospitalization time was evaluated, in the SDM group, the MDM group, and the SL group, it was found to be 611, 831, and 379 min, respectively. In multiparous pregnant women, the hospitalization time was 735 min in the SDM group, 494 min in the MDM group, and 261.5 min in the SL group (*p* < 0.05). Other than the hospitalization time, when the aforementioned variables were studied in multiparous pregnant women, no statistically significant difference among groups was observed (*p* > 0.05).

**Conclusion:**

Intravaginal misoprostol seems to be a promising medical agent for labor induction due to its high delivery rates within 12 hours and the absence of negative fetal outcomes, its ease of storage, and affordable cost.

## 1. Introduction

Labor induction is the initiation of contractions by mechanical or pharmacological stimulation of the uterus in order to achieve vaginal delivery [1]. Induction of labor is performed to initiate uterine contractions and ensure the dilatation of the cervix. The need for labor induction has increased in recent years with the increase in prenatal follow-up opportunities. The decision in relation to induction of labor should be made in cases where pregnancy continuation is not beneficial, or pregnancy continuation may be harmful from a maternal or fetal point of view. Indications for induction of labor can be summarized as follows: premature rupture of membranes, chorioamnionitis, fetal death, hypertensive diseases of pregnancy (preeclampsia and eclampsia), diabetes, renal disease, chronic pulmonary disease, logistic factors (psychosocial indications and distance from the hospital), and fetal growth restriction [2]. The rate of birth induction in nulliparous women in the United States of America (USA) in 2018 was reported as 37.8% [3].

Rapidly increasing cesarean section (C/S) rates have become a public health problem all over the world in recent years. In Turkey, this rate was determined as 51.9% between 2013 and 2016 and 51.2% in 2017 [4–6]. In addition to many nonmedical conditions (medico-legal problems and increased anxiety) that force both the clinician and pregnant women to have a cesarean delivery [7, 8], the absence of an absolutely effective method of induction of labor also increases C/S rates [9].

Labor induction methods are divided into two types: mechanical and pharmacological. Mechanical methods include sexual intercourse, nipple stimulation, membrane stripping, amniotomy, dilators, and balloon catheters [10]. Among the pharmacological methods, oxytocin, prostaglandin (PG) E2 (dinoprostone), prostaglandin E1 (misoprostol), and mifepristone (RU-486) are the most frequently used methods [11]. Although dinoprostone use has come to the fore in recent years, it creates problems for the clinician due to difficulty with respect to storage conditions, expensiveness, and risk of hyperstimulation [12]. Misoprostol has always been on the agenda because it is easy to use and inexpensive, can be used for postpartum bleeding, and has a higher birth rate than dinoprostone. But the issue that the clinician is most afraid of is the risk of hyperstimulation [12].

In this study, we compared the vaginal delivery rate within 12 hours, the total vaginal delivery rate, the duration of the second stage of labor, the need for interventional delivery, and neonatal APGAR scores among women who presented in the latent phase of labor and gave birth by labor induction with misoprostol or spontaneously.

## 2. Material and Method

Between June 2021 and December 2021, 213 pregnant women who were followed up in the Acibadem Maslak University Hospital for vaginal delivery were included in the study. Maternal and neonatal outcomes of pregnant women who had labor induction with intravaginal misoprostol and those who had spontaneous delivery were evaluated retrospectively from their records. Ethical approval was granted from the Medical Ethics Committee of the Institutional Ethical Review Board of the Acibadem Mehmet Ali Aydinlar University School of Medicine (number: 2022-07/37, date: 22 April 2022). All reported research was conducted in accordance with the principles set out in the Helsinki Declaration 2008. Pregnant women who were between 18 and 40 years of age, whose gestational week was between 37 and 41, and those with vertex presentation were included in the study. The age, gravida, parity, body mass index (BMI), and ultrasonography (USG) findings of the pregnant women before induction were recorded. Bishop score values obtained by vaginal examination at the time of admission were obtained retrospectively from the files of the patients [13]. Women were excluded if any of the following criteria were met: placenta previa, placenta abruption, breech or transverse presentation, significant cephalopelvic disproportion, suspected macrosomia, fetal distress, fetal congenital malformation, and prior uterine surgical procedure history [14]. Women with severe chronic diseases of the cardiac, pulmonary, hepatic, renal, hematopoietic, endocrine, or immune system; acute infection; cervical carcinoma; and contraindications for the use of PG analogues (glaucoma, asthma, epilepsy, and allergy to PG) were also excluded [15].

Indications for induction of labor were classified as maternal, fetal, elective, postmaturity, rupture of membranes, and term pregnancy with irregular contractions and dilatation. Maternal causes were preeclampsia, chronic hypertension, gestational diabetes, and logistic reasons (such as distance from hospital and risk of rapid labor). Fetal causes were fetal growth restriction, chorioamnionitis, and oligohydramnios. Elective labor induction was offered to pregnant women at 39 weeks of pregnancy to lower the frequency of cesarean delivery. Term pregnant women with >3 cm cervical dilatation but irregular uterine contractions, who underwent induction to achieve regular contractions and accelerate the transition to the active phase of labor, were evaluated in the term pregnant with irregular contraction group.

Every 4 hours, 25 mcg of misoprostol was applied to the posterior vaginal fornix. The protocol was based on the American College of Obstetricians and Gynecologists (ACOG) Bulletin No. 107 [16]. A vaginal examination was performed before each new dose administration. If the Bishop score was above 6, if the patient had regular uterine contractions, or if fetal monitoring was out of category I, no further misoprostol doses were administered. Our oxytocin protocol began with 2 milliunits/min of oxytocin (10 international units of oxytocin diluted in 1000 mL of 0.9 NaCl), which was increased by 2 milliunits/min every 30 minutes until uterine contraction frequency was every 2-3 minutes and contractions lasted 40-60 seconds, up to a maximum dose of oxytocin of 30 milliunits/min. During the process, pregnant women were followed up with a nonstress test (NST). During the NST follow-up period in active labor, category II NST and nonrecovery or category III NST follow-up were evaluated as fetal distress.

In cases where the second stage of labor needed to be accelerated, such as the mother's failure to push or the development of fetal distress, interventional delivery was performed by using the Kiwi® Complete Vacuum Delivery System (Laborie Medical Technologies Corp., Portsmouth, USA) or forceps.

The pregnant women, who gave birth, were divided into 3 groups as follows: spontaneous labor, those induced by a single dose of misoprostol, and those induced by multiple doses of misoprostol. The spontaneous labor group consisted of patients whose labor had started, no intervention such as misoprostol administration was made for cervical ripening, but induction with oxytocin was required at some stage of labor to accelerate it, or no intervention was made at all. The groups were compared in terms of delivery type, need for intervention, time spent in the second stage of labor, number of pregnant women with C/S due to fetal distress, amount of oxytocin if needed, need for epidural analgesia, and 1st and 5th minute neonatal APGAR scores.

### 2.1. Statistical Analysis

In the analysis of the variables, the SPSS 26.0 (IBM Corporation, Armonk, New York, United States) program was used. The conformity of the data to normal distribution was evaluated with the Shapiro-Wilk Francia test, while the homogeneity of variance was evaluated with the Levene test. The Mann–Whitney *U* test was used together with the Monte Carlo results to compare two independent groups according to quantitative variables that did not show normal distribution. The one-way ANOVA (robust test: Brown-Forsythe) test was used to compare more than two groups based on quantitative variables with a normal distribution, while the Kruskal-Wallis *H*-test Monte Carlo simulation results were used for quantitative variables with nonnormal distribution, and the Dunn's test was used for post hoc analysis. In the comparison of categorical variables with each other according to the groups, the Pearson chi-square and Fisher-Freeman-Halton tests were tested with the Monte Carlo simulation technique, and column ratios were compared with each other and demonstrated according to *p* value results with the Benjamini-Hochberg correction. Quantitative variables were expressed as mean (standard deviation) and median (minimum/maximum) in the tables, while categorical variables were shown as *n* (%). The variables were analyzed at a 95% confidence level, and a *p* value of less than 0.05 was considered significant.

## 3. Results

Two hundred and thirteen pregnant women, who met the inclusion criteria, were included in the study. Although misoprostol was used for labor induction in 118 (55.4%) of the patients, spontaneous labor was observed in 95 (44.6%) patients. The mean age of these pregnant women was 32.57 ± 4.21, with 110 (51.64%) pregnant women aged 24-28. While their mean BMI was 25.54 ± 3.90 kg/m^2^, 95 (44.6%) of them were in the ideal BMI range of 18.5 to 24.9 kg/m^2^. The gestational age of the pregnant women was 38.87 ± 1.15 weeks. The Bishop scores of the pregnant women, which were evaluated at the time of their first admission to the hospital, were 4.35 ± 2.25. The mean amount of misoprostol administered to all pregnant women participating in the study was 0.93 ± 1.04. The babies' birth weights were 3281.22 ± 319.28 grams (g). In addition to the aforementioned data, the 2nd stage of labor duration, the time passed from the last misoprostol application until birth, the 1st and 5th minute neonatal APGAR scores, and the difference between the 5th minute APGAR score and the 1st minute APGAR score are given in Table 1.

Of the pregnant women who participated in the study, 151 (70.9%) were primiparous, and 62 (29.1%) were multiparous. 57 (26.8%) pregnant women delivered with multiple doses of misoprostol, while 61 (28.6%) delivered with a single dose of misoprostol. For 101 (47.4%) pregnant women, oxytocin was needed to support contractions. Delivery of 149 (70.0%) pregnant women occurred within 12 hours of hospitalization. Fetal distress developed in 12 (5.6%) pregnant women. Vacuum extraction was performed in 26 (12.2%) deliveries, and forceps were used for 1 (0.46%) pregnant woman. 29 (13.6%) infants required NICU admission, and all infants were discharged upon recovery. In addition to these, the amount of epidural anesthesia, the gender of the baby, and the birth results are indicated in Table 2.

Age, BMI, gestational week, Bishop score, amount of dose administered, additional oxytocin need, indication of induction, need for epidural analgesia, duration of the second stage of labor, time from the last misoprostol dose administration to delivery, need for interventional delivery, fetal distress, gender of the baby, infant birth weight, NICU need, hospitalization time, difference parameters between 1st and 5th minute APGAR scores, and 5th and 1st minute APGAR scores of the pregnant women were evaluated among the groups, who received multiple doses and a single dose and went into spontaneous labor, both with respect to primiparous and multiparous pregnant women. The results are evaluated in Table 3.

Primiparous and multiparous pregnant women were grouped as those who had given birth in the first 12 hours and those who had not. A comparison was made to determine whether there was a difference between the SL, SDM, and MDM groups in terms of gestational week at birth, bishop score, and rates of induction of labor due to rupture of membranes in patients who had given birth within 12 hours and those who had not (Table 4).

## 4. Discussion

This study was carried out on pregnant women, who applied to our clinic in the latent phase of labor and delivered vaginally. We compared the results of the pregnant women, who gave birth with the help of either single or multiple doses of misoprostol and those who had spontaneous labor, retrospectively.

Labor induction methods were divided into two as follows: mechanical methods and pharmacological methods. In daily practice, oxytocin and misoprostol are the most commonly used pharmacological methods [17]. In recent years, dinoprostone has come into use. But the cost of dinoprostone and difficulty in storing it prevent its widespread use [12]. Therefore, pharmacological agents such as oxytocin and misoprostol remain relevant. In their study, Zhang et al. compared vaginal administration of 25 *μ*g misoprostol once every 4 hours (max 3 doses) with placebo drug administration [18]. At the 12th hour of induction, it was determined that a Bishop score ≥ 3 was achieved, the time between active labor and delivery was shortened, and the rate of delivery increased in the first 24 hours. However, no change was found in the median time to reach vaginal delivery, the incidence of C/S, and maternal and fetal adverse outcomes. In our study, 55.4% of the pregnant women gave birth with a single dose or multiple doses of misoprostol. In accordance with the literature, the effect of misoprostol on labor induction is indisputable, as supported by our study. The limitations of our study are that it was retrospective, and misoprostol was not compared with a placebo or a different induction agent. One of the important discussion topics in the literature is the safety of misoprostol use. The most important point, in this case, is the dosage. In a study by Pimentel et al., the rates of vaginal delivery as a result of a single 25 mcg dose of misoprostol and multiple 25 *μ*g doses of misoprostol administration once every 4-6 hours within 12 hours and 24 hours were compared [19]. C/S delivery rates were found to be similar between the groups. However, when the cases with C/S delivery were evaluated in the same study, it was observed that the rate of nulliparous pregnant women who gave birth by C/S increased in single-dose misoprostol administration, compared to multiple-dose misoprostol administration (49.3% and 28.6%, respectively). The biggest risk factor for C/S was the Bishop score being <4 and nulliparity. McMaster et al. reviewed 13 studies, in which 25 mcg and 50 mcg of intravaginal misoprostol were administered, as a meta-analysis [20]. Accordingly, the effectiveness of 25 mcg of misoprostol in terms of vaginal delivery was found to be less than that in the 50 mcg group (RR 0.59; 95% CI: 0.39–0.88). In the same meta-analysis, the rate of vaginal delivery within 24 hours was also found to be lower (RR 0.88; 95% CI 0.79–0.96) [20].

Our study was conducted on pregnant women who gave birth vaginally. There was no statistically significant difference between Bishop scores in both primiparous and multiparous groups, who received either single or multiple doses of misoprostol. As a result, we might assume that the Bishop score might not affect the outcomes of misoprostol administration in pregnant women, who are not in active labor. Among primiparous pregnant women, 65.2% of those who received a single misoprostol dose and 37% of those who received multiple misoprostol doses delivered vaginally within 12 hours. This difference is statistically significant. The same situation has not produced the same results in multiparous pregnant women. According to the results of our study, it was found that single-dose misoprostol administration was effective compared to multiple doses in primiparous pregnant women.

Another parameter we evaluated was the time between the last dose of misoprostol and delivery. When this period was compared between primiparous pregnant women, who received a single dose and multiple doses of misoprostol, the pregnant women who received a single dose of misoprostol had a statistically significant shorter period. The time from the last misoprostol dose to delivery, as well as the rate of vaginal delivery within 12 hours, did not comply with the general literature. In recent years, duration of hospitalization has become important for institutions (insurance companies, social security institutions, etc.) or individuals who cover health expenses. In our study, it was observed that a single dose of misoprostol administration instead of multiple doses reduced hospitalization time in primiparous pregnant women. Thus, given the importance of cost-effectiveness with respect to the method of induction, it is clear that more prospective large-scale studies are needed to increase the precision of these data.

Another curious aspect of misoprostol use is the way it is administered. In a study by Haas et al., vaginal misoprostol and buccal misoprostol administration were compared [21]. Misoprostol was administered to pregnant women at a dose of 25 mcg (first dose) and then 50 mcg (subsequent doses). There was no statistically significant difference in terms of the vaginal delivery rates between the vaginal and buccal misoprostol groups (84.2% vs. 77.4%, respectively). However, the duration of vaginal delivery was found to be shorter in the group given vaginal misoprostol (20.1 vs. 28.1 hours, respectively). The vaginal misoprostol group had a higher rate of vaginal delivery within 24 hours (58.6% vs. 39.2%, respectively) [21]. The dose of misoprostol required until active action was compared in the vaginal and buccal misoprostol administration groups. In terms of vaginal misoprostol, a statistically significant decrease was observed [21]. In our study, misoprostol was administered only vaginally.

Oxytocin may be required to support labor induction both when misoprostol is used and when spontaneous labor occurs [22]. In a study by Haas et al., the maximum dose of oxytocin administered after misoprostol was evaluated. The maximum oxytocin dose requirement was found to be statistically lower in the vaginally administered misoprostol group when compared to that in the buccal misoprostol group (4.0 vs. 6.0 median, respectively) [21]. In a meta-analysis by McMaster et al., it was observed that 25 mcg of misoprostol administration showed an increased oxytocin requirement compared to 50 mcg of oxytocin (RR 0.88; 95% CI 0.79–0.96) [20]. In a study by Pimentel et al., a statistical increase in maximum dose oxytocin use was observed in the group given single-dose misoprostol [19]. In our study, it was observed that the number of pregnant women, who required oxytocin, increased statistically in the group administered multiple doses of misoprostol among primiparous pregnant women. This situation does not correspond to that seen in the literature.

In our study, no statistically significant difference was observed between the groups in terms of fetal distress rate. In fact, fetal distress was not observed in any of the multiparous pregnant women, who were administered either a single dose or multiple doses of misoprostol, while fetal distress developed at a rate of 5.6% in those who were followed up with spontaneous delivery. In a study by Zhang et al., fetal distress suspicion was found in 47.1% of pregnant women. The rate of fetal heart rate abnormality was found to be 2.9% in the same study [18]. In a study by Kramer et al., abnormal FHR patterns were discovered in 29.6% of pregnant women during intrapartum NST follow-up. Late decelerations were found in 3% of the study group and prolonged decelerations in 6% [22].

Neonatal outcomes are a parameter that should be evaluated in terms of the safety of the method used for labor induction [23]. In the literature, many parameters such as APGAR score, umbilical cord pH analysis, and NICU admission are used to evaluate neonatal outcomes. In a study by Haas et al., the C/S ratio was found to be higher in the group administered buccal misoprostol due to fetal stress [21]. In a meta-analysis by McMaster et al., the NICU hospitalization rate of newborns in the 25 mcg group and the 50 mcg group was found to be 5.3% and 8.6%, respectively [20]. The number of newborns with an APGAR score < 7 was found to be 1.9% and 3.1% in the 25 mcg group and the 50 mcg group, respectively. In the evaluation of babies with meconium, it was found to be 9% and 13.3% in the 25 mcg group and the 50 mcg group, respectively [20]. In a study by Pimental et al., the number of babies with apH < 7.1in fetal umbilical cord pH analysis was evaluated [19]. Accordingly, it was found to be 1.8% and 5.2% in the 25 mcg group and the 50 mcg group, respectively. This difference is not statistically significant. Similar results apply to admission to the NICU inpatient unit. In our study, no statistically significant difference was found between all groups in terms of NICU hospitalization and the 1st and 5th minute APGAR scores. pH analysis was not performed in our study, which is another limitation.

When compared to pregnant women, who went into spontaneous labor, it is seen that the induction of labor with misoprostol does not adversely affect newborn outcomes. In primiparous pregnant women, single-dose misoprostol administration was found to be more effective than multiple-dose misoprostol administration in terms of oxytocin requirement, time from the last misoprostol administration to delivery, and hospitalization time. In multiparous pregnant women, there was no difference in misoprostol administration in terms of single-dose and multiple-dose administration.

## 5. Conclusion

Although prospective randomized studies are needed, considering the results we obtained in our study, we can say that misoprostol is a promising medical agent for labor induction with its high delivery rates in 12 hours without causing negative fetal outcomes, its ease of storage, and affordable cost.

## Figures and Tables

**Table 1:** Demographic characteristics of the study groups.

	*N*	Mean ± SD	Median (min-max)
Age (y)	213	32.57 ± 4.21	32 (24-43)
BMI (kg/cm^2^)	213	25.54 ± 3.90	25.31 (13.76-38.41)
Gestational week (week)	213	38.97 ± 0.83	39.1 (37-41)
Bishop score (*n*)	213	4.35 ± 2.25	4 (0-12)
Number of doses administered (*n*)	213	0.93 ± 1.04	1 (0-4)
Duration of stage 2 (min)	213	38.58 ± 30.55	31 (3-209)
Time passed from the last dose until delivery (min)	118	623.41 ± 359.53	600 (17-2880)
1st min APGAR (*n*)	213	8.82 ± 0.55	9 (6-10)
5th min APGAR (*n*)	213	9.69 ± 0.82	10 (8-10)
Difference APGAR (5-1) (*n*)	213	0.87 ± 0.77	1 (0-10)
Baby weight (g)	213	3281.22 ± 319.28	3270 (2201-4130)
Duration of hospitalization to birth (min)	213	600.96 ± 395.89	541 (11-2895)

SD: standard deviation.

**Table 2:** Demographic characteristics of subgroups.

	*n* (%)
Cytotec groups	
Multiple doses	57 (26.8)
Single dose	61 (28.6)
Spontaneous birth	95 (44.6)
Gravida	
Primiparous	151 (70.9)
Multiparous	62 (29.1)
Indication for induction	
Elective	60 (28.2)
Fetal	1 (0.5)
Term pregnant women with irregular contractions	87 (40.8)
Maternal	1 (0.5)
Postmaturity	6 (2.8)
Rupture of membranes	58 (27.2)
Oxytocin support	
No	112 (52.6)
Yes	101 (47.4)
Use of epidural analgesia	
No	49 (23.0)
Yes	164 (77.0)
Delivery within 12 hours	
No	64 (30.0)
Yes	149 (70.0)
Interventional delivery	
No	186 (87.3)
Yes	27 (12.7)
Type of intervention	
Forceps extraction	1 (3.7)
Vacuum extraction	26 (96.3)
Fetal distress	
No	201 (94.4)
Yes	12 (5.6)
Gender of the baby	
Female	103 (48.4)
Male	110 (51.6)
NICU (neonatal intensive care unit) admission	
No	184 (86.4)
Yes	29 (13.6)

**Table 3:** Comparison of the results of the subgroups.

	Primiparous			*p*	Multiparous			*p*
	Multiple (*n* = 46)	Single (*n* = 46)	Spontaneous (*n* = 59)		Multiple (*n* = 11)	Single (*n* = 15)	Spontaneous (*n* = 36)	
Age (y)	31.76 ± 3.92	31.70 ± 3.35	31.19 ± 3.80	0.677^a^	36.55 ± 3.67	35.60 ± 4.24	34.50 ± 4.53	0.315^a^
BMI (kg/cm^2^)	26.18 ± 4.11	25.31 ± 3.49	25.66 ± 4.24	0.566^a^	27.00 ± 4.80	24.28 ± 2.96	24.88 ± 3.52	0.209^a^
Gestational week	39.2 (37.5-41)	39.2 (37.3-40.3)	39.1 (37.1-40.4)	0.073^k^	39.1 (37.3-40.3)	38.5 (37.2-40.3)	39.2 (37.2-40.2)	0.519^k^
Bishop score	3 (2-6)	3 (0-6)	5 (2-12)^AB^	<0.001^k^	3 (2-5)	4 (2-7)	6 (3-12)^AB^	<0.001^k^
Number of doses administered	2 (2-4)^BC^	1 (1-1)^C^	0 (0-0)	<0.001^k^	2 (2-4)	1 (1-1)	0 (0-0)^AB^	<0.001^k^
Oxytocin initiation				0.021^C^				0.526^f^
No	15 (32.6)	25 (54.3)^A^	35 (59.3)^A^		7 (63.6)	7 (46.7)	23 (63.9)	
Yes	31 (67.4)^BC^	21 (45.7)	24 (40.7)		4 (36.4)	8 (53.3)	13 (36.1)	
Indication for induction of labor				<0.001^ff^				0.002^ff^
Elective	31 (67.4)^BC^	10 (21.7)^C^	4 (6.8)		6 (54.5)^C^	5 (33.3)	4 (11.1)	
Fetal indications	0 (0.0)	1 (2.2)	0 (0.0)		0 (0.0)	0 (0.0)	0 (0.0)	
Term pregnant women with irregular contractions	6 (13.0)	12 (26.1)	37 (62.7)^AB^		1 (9.1)	6 (40.0)	25 (69.4)^AB^	
Maternal indications	0 (0.0)	0 (0.0)	0 (0.0)		1 (9.1)	0 (0.0)	0 (0.0)	
Post maturity	3 (6.5)	1 (2.2)	0 (0.0)		0 (0.0)	1 (6.7)	1 (2.8)	
RoM	6 (13.0)	22 (47.8)^A^	18 (30.5)^A^		3 (27.3)	3 (20.0)	6 (16.7)	
Epidural analgesia				0.001^C^				0.090^ff^
No	3 (6.5)	5 (10.9)	19 (32.2)^AB^		2 (18.2)	3 (20.0)	17 (47.2)	
Yes	43 (93.5)^C^	41 (89.1)^C^	40 (67.8)		9 (81.8)	12 (80.0)	19 (52.8)	
Duration of second stage of labor (min)	36.5 (11-209)	33.5 (7-107)	34 (3-173)	0.792^k^	27 (15-180)	20 (5-119)	20.5 (4-141)	0.249^k^
Time from the last dose to delivery	720 (17-2880)	483 (60-1860)	—	<0.001^U^	600 (350-1500)	434 (241-756)	—	0.244^U^
Interventional delivery				0.609^C^				0.071^ff^
No	37 (80.4)	38 (82.6)	52 (88.1)		10 (90.9)	13 (86.7)	36 (100.0)	
Yes	9 (19.6)	8 (17.4)	7 (11.9)		1 (9.1)	2 (13.3)	0 (0.0)	
Fetal distress				0.999^ff^				0.999^ff^
No	43 (93.5)	43 (93.5)	55 (93.2)		11 (100.0)	15 (100.0)	34 (94.4)	
Yes	3 (6.5)	3 (6.5)	4 (6.8)		0 (0.0)	0 (0.0)	2 (5.6)	
Gender of the baby				0.836^C^				0.492^ff^
Female	19 (41.3)	22 (47.8)	26 (44.1)		5 (45.5)	8 (53.3)	23 (63.9)	
Male	27 (58.7)	24 (52.2)	33 (55.9)		6 (54.5)	7 (46.7)	13 (36.1)	
NICU admission				0.091^C^				0.999^ff^
No	42 (91.3)	36 (78.3)	54 (91.5)		9 (81.8)	13 (86.7)	30 (83.3)	
Yes	4 (8.7)	10 (21.7)	5 (8.5)		2 (18.2)	2 (13.3)	6 (16.7)	
Birth weight	3325.13 ± 309.42	3260.78 ± 342.12	3257.75 ± 306.05	0.507^a^	3289.09 ± 363.99	3177.67 ± 354.72	3330.42 ± 298.92	0.372^a^
Duration of hospitalization to birth (hours)	831.5 (515-2895)^BC^	611 (11-1920)^C^	379 (23-1466)	<0.001^k^	735 (385-1754)	494 (256-912)	261.5 (20-950)^AB^	<0.001^k^
APGAR								
1st min	9 (7-10)	9 (7-10)	9 (7-10)	0.333^k^	9 (8-10)	9 (8-9)	9 (6-10)	0.883^k^
5th min	10 (8-19)	10 (9-10)	10 (8-10)	0.816^k^	10 (9-10)	10 (9-10)	10 (8-10)	0.421^k^
The *p* value for APGAR 1st vs. 5th min (M)	<0.001^W^	<0.001^W^	<0.001^W^		0.008^W^	<0.001^W^	<0.001^W^	
Difference (5-1) m	1 (0-10)	1 (0-2)	1 (0-1)	0.725^k^	1 (0-1)	1 (0-1)	1 (0-2)	0.718^k^

^a^One-way ANOVA (robust statistic: Brown-Forsythe). ^k^Kruskal-Wallis test (Monte Carlo); post hoc test: Dunn's test. ^ff^Fisher-Freeman-Halton (Monte Carlo). ^e^Pearson chi-square test (Monte Carlo); post hoc test: Benjamini-Hochberg correction. ^U^Mann–Whitney *U* test (Monte Carlo). ^W^Wilcoxon signed rank test (Monte Carlo); quantitative variables are shown as mean ± SD or median (min-max); categorical data were shown as *n* (%). ^A^Significance according to the multiple-dose group. ^B^Significance according to the single-dose group. ^C^Significance according to the spontaneous birth group.

**Table 4:** Comparison of those who gave birth within 12 hours and who did not.

	Primiparous			*p*	Multiparous			*p*
	Multiple (*n* = 46)	Single (*n* = 46)	Spontaneous (*n* = 59)		Multiple (*n* = 11)	Single (*n* = 15)	Spontaneous (*n* = 36)	
Delivery within 12 hours				<0.001^C^				0.185^ff^
No	29 (63.0)^BC^	16 (34.8)^C^	9 (15.3)		4 (36.4)	2 (13.3)	4 (11.1)	
Yes	17 (37.0)	30 (65.2)^A^	50 (84.7)^AB^		7 (63.6)	13 (86.7)	32 (88.9)	
Gestational week at birth								
No^∗^	39.2 (37.5-37.5)^C^	38.5 (36.4-36.4)	38.4 (35.4-35.4)	0.006^k^	38.6 (37.3-37.3)	40.2 (40.1-40.1)^excluded^	37.85 (35.6-35.6)	0.912^U^
Yes^∗∗^	39.2 (38.1-38.1)	39.25 (35.4-35.4)	39.2 (35.6-35.6)	0.290^k^	39.3 (37.4-37.4)	38.4 (30.4-30.4)^excluded^	39.2 (37.2-37.2)	0.709^U^
Bishop score								
No^∗^	3 (2-2)	3 (0-0)	4 (3-3)^AB^	0.002^k^	4.5 (2-2)	3.5 (3-3)	6 (3-3)	0.257^k^
Yes^∗∗^	3 (2-2)	3 (2-2)	5 (2-2)^AB^	<0.001^k^	3 (2-2)	4 (2-2)	6 (3-3)^AB^	<0.001^k^
Indication for induction (RoM)				0.736^f^^f^				0.999^ff^
No^∗^	2 (33.3)	7 (31.8)	4 (22.2)		0 (0.0)	0 (0.0)	1 (16.7)	
Yes^∗∗^	4 (66.7)	15 (68.2)	14 (77.8)		3 (100.0)	3 (100.0)	5 (83.3)	

^a^One-way ANOVA (robust statistic: Brown-Forsythe). ^k^Kruskal-Wallis test (Monte Carlo); post hoc test: Dunn's test. ^ff^Fisher-Freeman-Halton (Monte Carlo). ^e^Pearson chi-square test (Monte Carlo); post hoc test: Benjamini-Hochberg correction. ^U^Mann–Whitney *U* test (Monte Carlo). ^W^Wilcoxon signed rank test (Monte Carlo); quantitative variables are shown as mean ± SD or median (min-max); categorical data were shown as *n* (%). ^A^Significance according to the multiple-dose group. ^B^Significance according to the single-dose group. ^C^Significance according to the spontaneous birth group. ^∗^Pregnant women who did not deliver in 12 hours. ^∗∗^Pregnant women who delivered in 12 hours.

## Data Availability

The datasets used for the current study are available from the corresponding author, per reasonable request.
